# Clinical Use and Mechanisms of Infliximab Treatment on Inflammatory Bowel Disease: A Recent Update

**DOI:** 10.1155/2013/581631

**Published:** 2013-01-21

**Authors:** Yuan Guo, Nonghua Lu, Aiping Bai

**Affiliations:** ^1^Department of Gastroenterology, The First Affiliated Hospital of Nanchang University, Nanchang 330006, China; ^2^Department of Pharmacy, The First Affiliated Hospital of Nanchang University, Nanchang 330006, China

## Abstract

The pathogenesis and treatment of inflammatory bowel disease (IBD) have been recently advanced, while it is still challenged with high morbidity and poor prognosis. Infliximab, a monoclonal antibody of tumor necrosis factor (TNF), has emerged as an efficient treatment with many clinical benefits such as quick disease activity reduction and IBD patient life quality improvement. However, the biological effects of infliximab on IBD need to be elucidated. This paper reviewed the clinical use and recently advanced biological action of infliximab on IBD. By forming the stable complex with the soluble or the membrane form of TNF in fluid environment or on cell surface of immune cell, fibroblast, endothelium, and epithelium, infliximab quenches TNF activity and performs the important biological actions which lead to amelioration and remission of immune responses. The mechanisms of infliximab treatment for IBD were intensively discussed. The recent advances on two topics including predictors and side effects of infliximab treatment were also reviewed.

## 1. Introduction

Inflammatory bowel disease (IBD), mainly containing Crohn's disease (CD) and ulcerative colitis (UC), is a group of chronic inflammatory disorders in the colon and small intestine. Although the etiology of IBD remains unclear, the pathogenesis of IBD has been recently advanced. It is strongly suggested that altered immunological function, resulting from an interplay between genetic susceptibility and certain environmental factors including bacteria infection, contributes to the development of mucosal inflammatory responses of gastrointestinal tract [1]. Proinflammatory cytokines, especially tumor necrosis factor (TNF), are produced mainly by activated immune cells in inflamed mucosa during the process of IBD, and those proinflammatory cytokines further activate immune cells, as the feedback, to produce toxic molecules including super oxygen products, chemokines, proteinases, and cytokines which result in tissue damage and inflammation development [2, 3]. 

In the past years, TNF has been known to play a pivotal role in the pathogenesis of IBD [4]. When released by active macrophages and T lymphocytes, TNF initiates multiple biological reactions below: modulates immune cell function, drives adaptive immune responses, triggers epithelium apoptosis and breaks epithelial barrier, induces endothelium expressing adhesion molecules such as intercellular adhesion molecule 1 (ICAM1) to recruit immune cells, and regulates matrix metalloproteinase (MMP) expression to induce tissue degradation and damage [5, 6]. Clinical studies have shown that TNF protein and mRNA levels are elevated in serum, intestinal tissue, stool of active IBD, in correlation with disease activity [4, 7–9]. Clinical inhibition of TNF production has been linked with disease remission, improved life quality, and relapse prevention, meanwhile, failure of clinical treatment of IBD has been attributed to early reactivation of TNF secretory capacity by immune cells [10, 11]. The findings suggest that TNF is critical for disease development. Inhibiting TNF production in inflamed mucosa is one of the important goals for IBD management.

The conventional treatments of IBD include corticosteroids and aminosalicylates. However, only 50% of patients achieve sustained remission with the conventional drugs which can raise many side effects [12]. Recently, many novel drugs have been developed for clinical IBD management, and among them, TNF neutralization by monoclonal antibodies has been shown as one of the effective approaches for IBD treatment [13].

### 1.1. TNF and Infliximab

TNF is primarily a type II transmembrane protein with 212 amino acid sequence and exists in a stable homotrimer. TNF alpha converting enzyme (TACE) is a metalloprotease which can cleave membrane-integrated TNF and release TNF in a soluble homotrimeric form [4, 9]. Both membrane and soluble TNF can perform their biological function by binding to their receptors including receptor 1 (TNFR1, CD120a) and TNFR2 (CD120b), which are expressed by most tissue cells. Through its receptor TNFR1, TNF can activate intracellular pathways including transcription factor nuclear factor-*κ*B (NF-*κ*B) and mitogen-activated protein kinase (MAPK), which are key kinases in regulating immune cell activation and migration, inducing gene expression of chemokines, cytokines, and toxic molecules including reactive oxygen species [4, 9]. 

The technique of TNF blockade by monoclonal antibodies has been developed as an effective therapy for immune diseases including IBD [14, 15]. As the first monoclonal TNF antibody approved for human treatment, infliximab is a purified, recombinant DNA-derived chimeric human-mouse IgG monoclonal antibody and contains murine heavy (H) and light (L) chain variable regions (VH and VL, resp.), ligated to genomic human heavy and light chain constant regions [14, 16]. Infliximab can quickly form stable complexes with the human soluble or the membrane form of TNF and terminate the biological activity and signals of TNF [11]. With a serum half-life of 9.5 days and still detectable in serum of IBD patients 8 weeks after infusion treatment, infliximab provides a useful strategy to neutralize TNF and to inhibit immune responses of IBD [17]. After approved by the Food and Drug Administration of USA (FDA) in 1998, infliximab has been used for IBD treatment for about 15 years, and its clinical efficacy has been well studied.

## 2. Clinical Use of Infliximab for IBD Treatment

At the beginning of clinical application, infliximab had been used for Crohn's disease patients with intestine inflammation. Targan et al. conducted a 12-week multicenter, double-blind, placebo-controlled trial of cA2 (infliximab) in Crohn's disease patients [50]. 108 moderate-to-severe patients resistant to steroid randomly conducted a single two-hour intravenous infusion of either placebo or cA2 in a dose of 5 mg/kg body weight, 10 mg/kg, or 20 mg/kg. At four weeks, 22 of 27 patients given 5 mg/kg, 14 of 28 given 10 mg/kg, and 18 of 28 given 20 mg/kg had had a clinical response, as compared with 4 of 24 patients in the placebo group (*P* < 0.001 for the comparison of the cA2 group as a whole with placebo). 33% patients given cA2 went into remission, as compared with 4% of the patients given placebo (*P* = 0.005). At 12 weeks, 34 of 83 cA2-treated patients had had a clinical response, as compared with 3 of 25 patients in the placebo group (*P* = 0.008). Infliximab for Crohn's disease remission maintenance was also reported [51].

Besides clinical use for intestinal inflammation, infliximab has currently been authorized for another two phenotypes of active Crohn's disease: stricturing disease (which causes narrowing of the bowel) and penetrating disease (which causes fistulae or abnormal connections of the bowel) [52–54]. In a multicenter, double-blind, randomized, placebo-controlled trial [55], 306 Crohn's disease patients with one or more draining abdominal or perianal fistulas of at least three months' duration received infliximab (5 mg/kg) intravenously on weeks 0, 2, and 6. A total of 195 patients who had a response at weeks 10 and 14 and 87 patients who had no response were then randomly assigned to receive placebo or infliximab every eight weeks and to be followed to week 54. The time to loss of response was significantly longer for patients who received infliximab maintenance therapy than for those who received placebo maintenance (more than 40 weeks versus 14 weeks, *P* < 0.001). At week 54, 19 percent of patients in the placebo maintenance group had a complete absence of draining fistulas, as compared with 36 percent of patients in the infliximab maintenance group (*P* = 0.009). As shown by this and other trials, infliximab was effective in short-term closure of rectovaginal fistulas and maintenance treatment was more effective than placebo in prolonging rectovaginal fistula closure.

Clinical efficacy of infliximab on ulcerative colitis has been clarified. Many clinical trials evaluated the effect of infliximab as induction and maintenance therapy for the patients with ulcerative colitis. In two randomized, double-blind, placebo-controlled studies - the Active Ulcerative Colitis Trials 1 and 2 (ACT 1 and ACT 2, resp.,) [56], 364 patients with moderate-to-severe active ulcerative colitis were given placebo or infliximab (5 mg or 10 mg/kg) intravenously at weeks 0, 2, and 6 and then every eight weeks through week 46 (in ACT 1) or week 22 (in ACT 2). In ACT 1, 69 percent of patients who received 5 mg of infliximab and 61 percent of those who received 10 mg had a clinical response at week 8, as compared with 37 percent of those who received placebo (*P* < 0.001 for both comparisons with placebo). In ACT 2, 64 percent of patients who received 5 mg of infliximab and 69 percent of those who received 10 mg had a clinical response at week 8, as compared with 29 percent of those who received placebo (*P* < 0.001 for both comparisons with placebo). In both studies, patients who received infliximab were more likely to have a clinical response at week 30 (*P* ≤ 0.002 for all comparisons). In ACT 1, more patients who received 5 mg or 10 mg of infliximab had a clinical response at week 54 (45 percent and 44 percent, resp.,) than did those who received placebo (20 percent, *P* < 0.001 for both comparisons). Recently, other studies further showed that infliximab is an alternative treatment for induction and maintenance therapy in moderately to severely active ulcerative colitis [57].

## 3. Mechanism of Infliximab Treatment on Inflammatory Bowel Disease

In soluble or transmembrane form, TNF plays multiple functions in the process of IBD. By blocking and neutralizing TNF activity, infliximab has shown its high effectiveness in the clinical management of Crohn's disease and ulcerative colitis. Up to now, biological action of infliximab on IBD has been explored (Table 1).

### 3.1. Inhibiting Inflammatory Cytokines and Mediators

During the process of IBD, TNF is highly produced by immune cells, and as the feedback, TNF recruits immune cells to move from blood vessel into inflammation sites and induces the cells to release inflammatory cytokines and mediators [58, 59]. Inhibiting TNF production by infliximab can downregulate the expression of inflammatory cytokines and mediators in intestinal tissues and ameliorate IBD [18, 19]. Ljung et al. studied the time-course of the effects of infliximab with reference to mucosal cytokine and inducible nitric oxide synthase expression [20]. Thirty-two patients with Crohn's disease were involved in infliximab treatment, and clinical response was seen in 14 patients with downregulation of global immunohistochemistry expression, reaching nadir day 3. Immunohistochemical staining showed that rectal nitric oxide was increased at baseline in patients compared with control people. After 28 days treatment, decreased rectal nitric oxide levels parallel downregulation of inducible nitric oxide synthase, TNF, IFN-*γ*, and IL-1*β* levels in the patients with clinical response. 

Olsen and other researchers found that infliximab therapy decreased the levels of TNF and IFN-*γ* mRNA in colonic mucosa of ulcerative colitis patients [60]. Thirty-two patients with active ulcerative colitis received infliximab therapy showed clinical and endoscopic improvements. Infliximab reduced the expression of TNF and IFN-*γ* mRNA, but not that of IL-10 and IL-4 mRNA in mucosa of ulcerative colitis patients. Reductions in TNF mRNA were correlated to clinical and endoscopic improvements. 

Granulocyte-macrophagecolony-stimulating factor (GM-CSF) is linked with the pathogenesis of IBD [61]. Agnholt and colleagues studied the effect of infliximab treatment on GM-CSF production in Crohn's disease patients [21]. Biopsies from Crohn's disease patients before and after infliximab administration and from ten healthy subjects were cultured, and GM-CSF content was analyzed after 5 days. GM-CSF production was increased in Crohn's disease patients compared with healthy controls and correlated with Crohn's disease activity index. GM-CSF levels and mucosal histology score were decreased after three infliximab infusions, indicating the pivotal role of infliximab treatment in controlling GM-CSF production, inflammation, and disease activity in Crohn's disease.

### 3.2. Modulating Immune Cell Functions

Excessive activation of innate and adaptive immune cells contributes to persistent development of IBD [62, 63]. Moreover, mucosal T cells show resistance to activation-induced apoptosis in Crohn's disease, leading to accumulation of T cells in mucosa and large amount of cytokines production [64]. One action of infliximab treatment is to induce immune cell apoptosis by outside-to-inside signaling through transmembrane TNF (mTNF). Van den Brande et al. cultured peripheral blood lymphocytes and lamina propria T cells from patients with Crohn's disease or from healthy volunteers and treated the cells with infliximab or etanercept, two TNF-neutralizing drugs, respectively [22]. By using fluorescence-activated cell sorter analysis, annexin V staining, and cleaved caspase-3 immunoblotting, they found that infliximab bound to peripheral blood lymphocytes and lamina propria T cells from patients with Crohn's disease, and subsequently induced apoptosis of activated lymphocytes, compared with those from healthy volunteers, whereas etanercept did not show the same effect. Other studies had been performed to verify the in vivo effect of infliximab on mucosal T cell death from Crohn's disease. In a pilot study, 10 Crohn's disease patients received three consecutive infusions of infliximab, endoscopic intestinal biopsies were collected after 10 weeks of treatment. As detected by TUNEL assay, infliximab treatment induced a sustained mucosal T apoptosis in Crohn's disease patients [23]. Another group also found that infusion of infliximab in steroid refractory patients with Crohn's disease induced a significant increase in CD3 and TUNEL positive cells within colonic biopsies, and those infliximab-stimulated apoptotic cells were activated T lymphocytes but not resting cells [24].

IBD is characterized by imbalance T helper cell (Th) responses, with dominance of Th1 and Th17 responses while deficiency of Th2 and Treg responses [65–67]. Downregulating the established Th1 cell responses, or shifting to Th2 and Treg responses, can successfully ameliorate IBD, as indicated by clinical and experimental research [25–27]. Agnholt and Kaltoft studied in vitro the effect of infliximab on lamina propria T cell responses [28]. Mucosal T cell cultures from patients with Crohn's disease produced significantly higher levels of IFN-*γ* and TNF than T cell cultures from healthy controls, while the production of IFN-*γ* was downregulated by infliximab treatment in early T cell cultures from Crohn's disease patients. Matsumura et al. have recently shown that 2 weeks infliximab infusion therapy can also modulate Th1 and Th2 balance of active ulcerative colitis and induce remission of the ulcerative colitis [68].

Infliximab therapy has been recently shown that it can modulate regulatory T cell (Treg) response in mucosal of IBD patients. Inappropriate immune responses including dominant Th1 and Th17, while deficient Treg contributes to the progress of the intestinal immune system in IBD. Increased apoptosisofTregwas detected in IBD, suggesting that a numerical deficiency ofTreg responds to an insufficient compensation of chronically activatedT lymphocytes [29]. Recent studies have been reported that infliximabtreatmentrapidly increases the frequency of functional Treg in active IBD, affects Treg activation and expansion, and enhances the suppressive function of Treg [29, 30]. 

Regulatorymacrophage is a subtype innate immune cell with anti-inflammatory properties, appears to modulate inflammatory immune responses, and plays an important role in wound healing and gut homeostasis [31]. The effect of infliximab treatment on regulatory macrophage induction has recently been explored. Hommes and colleagues have shown that the antibodies against TNF including infliximab and adalimumab induce formation of regulatorymacrophagesin an Fc region-dependent manner. The induced regulatorymacrophages inhibit proliferation of activated T cells, produce anti-inflammatory cytokines, and express the regulatory macrophagemarker CD206 [31]. 

### 3.3. Protecting Intestinal Epithelial Cells from Apoptosis

Loss of epithelial barrier integrity, characterized with increased apoptosis of epithelia, is considered an early step in the pathogenesis of IBD [32]. TNF, as the major proinflammatory mediators in IBD, is one of the extrinsic signals which initiate apoptosis of enterocytes [69]. The action of infliximab treatment is linked with protecting intestinal epithelial cells from apoptosis. A clinical study presented that epithelial apoptoses were upregulated in the colon in Crohn's disease and restored to normal in majority of patients by infliximab treatment [33], which correlated with epithelial barrier dysfunction measured as epithelial resistance.

Enterocyte apoptosis contributes to intestinal barrier dysfunction and initiation of intestinal inflammation [70]. Suenaert and colleagues studied intestinal permeability in refractory Crohn's disease before and after treatment with infliximab [32]. 4 weeks infliximab infusion decreased intestinal permeability of active Crohn's disease, measured by urinary excretion of [32] Cr-EDTA after oral intake, confirming the essential role of TNF in modulating gut barrier in IBD. 

### 3.4. Restoring Endothelium Function and Blocking Angiogenesis

Endothelium has multiple functions such as forming the semiselective barrier between the vessel lumen and surrounding tissue, controlling the passage of materials and infiltration of imune cells into tissues. During the process of IBD, the expressions of leukocyte/endothelial cell adhesion molecules such as intercellular adhesion molecule 1 (ICAM1) and vascular cell adhesion molecule 1(VCAM1) were upregulated, which recruit leukocyte migration into intestinal tissue, as shown by the Affymetrix gene expression microarray study [34]. After infliximab infusion, the upregulated leukocyte cell adhesion molecules were restored, in parallel with the disappearance of the inflammatory cells from the colonic lamina propria. Also, endothelial cell adhesion molecules and most chemokines/chemokine receptors in colon tissues returned to normal after infliximab therapy. The findings support the concept that infliximab regulates immune cell infiltration by controlling leukocyte/endothelial cell adhesion molecules and chemokines/chemokine receptors.

Endothelial dysfunction was reported in patients with IBD, and attributed to TNF produced in intestine. Schinzari et al. found that endothelium-dependent vasodilation to acetylcholine was impaired inCrohn's disease, but not in ulcerative colitis. TNF neutralizing treatment with infliximab enhanced the responsiveness of endothelium to acetylcholine inCrohn's disease patients, indicating that endothelial function is impaired inCrohn's diseaseand is beneficially restored by infliximab treatment [35].

Angiogenesishas been shown a critical component of neoplastic and chronic inflammatory disorders. Recently, local microvasculature was reported to be triggered by vascular endothelial growth factor A (VEGF-A) [36], and feedback to undergo an intense process of inflammation-dependentangiogenesis, and contribute to IBDpathogenesis [71]. Scaldaferri et al. have recently reported that the expression of proliferation marker Ki-67 in endothelial cells was significantly reduced by infliximab administration, in concomitance with decreased mucosal concentration of VEGF-A [36]. The data indicate another novel effect of infliximabtreatment in downregulating mucosalangiogenesisin IBD and restraining production of VEGF-A.

### 3.5. Regulating the Balance between Matrix Metalloproteinases (MMPs) and Tissue Inhibitor of Metalloproteinases (TIMPs)

Matrix metalloproteinases (MMPs) and their endogenous inhibitors, tissue inhibitors of MMPs (TIMPs), are produced in the gastrointestinal tract by several structural cells, including neutrophils, platelets, mesenchymal cells, T cells, monocytes, macrophages, and cancer cells [72]. The balance between MMPs and TIMPs is essential for many physiological processes in the gut. However, imbalance between MMPs and TIMPs plays an important role in the pathophysiology of diverse intestinal inflammatory conditions including IBD [73]. Infliximab treatment can restore the balance between MMPs and TIMP seen in IBD.

Meijer et al. determined expression and secretion of MMP-1, -2, -3, -9, and their inhibitors TIMP-1, -2 by IBD versus control intestinal mucosa ex vivo and to assess the regulatory capacity by infliximab of the proteolytic phenotype [37]. Intestinal mucosal explants from 20 IBD and 15 control patients were cultured with or without infliximab and/or the T-cell activator pokeweed mitogen (PWM). Expression of MMP and TIMP protein/activity in basal medium was higher in IBD versus control explants. infliximab downregulated MMP-1, -3, and -9 relative to TIMP-1 and -2 and also decreased MMP-1 and -3 activities, while PWM enhanced these levels, partly counteracted again by infliximab. The expression of MMP-2 relative to TIMP did not change by treatment with infliximab and/or PWM.

Di Sabatino et al. investigated the action of infliximab on apoptosis, the production of matrix metalloproteinases (MMPs) and tissue inhibitor of metalloproteinases (TIMPs)-1, and migration of Crohn's disease myofibroblasts [38]. Colonic myofibroblasts were isolated from patients with active Crohn's disease and controls, and treated by infliximab. Crohn's disease myofibroblasts showed higher mTNF expression than control myofibroblasts. Infliximab had no effect on Crohn's disease myofibroblast apoptosis, caspase-3 activation, and production of MMP-3 and MMP-12. However, infliximab induced a significant dose-dependent increase in TIMP-1 production. The migration of Crohn's disease myofibroblasts was enhanced significantly by infliximab and recombinant human TIMP-1, and infliximab-induced migration was inhibited by anti-TIMP-1 neutralizing antibody. Infliximab also decreased Crohn's disease myofibroblast collagen production. Together, infliximab therapy can enhance TIMP-1 production and myofibroblast migration, while reduce MMP activity and facilitate the wound healing.

### 3.6. Improving Mucosal Healing

Mucosal healing has appeared as an important treatment goal for patients with IBD, and can alter the course of IBD with sustained clinical remission and reduced rates of hospitalization and surgical resection [74]. Recently, infliximab treatment has emerged as a therapy for mucosal healing induction. Kierkus et al. have recently evaluated the impact of infliximab infusion on induction of intestinal mucosal healing [39]. 66 pediatric patients with Crohn's disease were involved in the study with infliximab infusion. Healing induction therapy with infliximab was found to be clinically effective in 72% of pediatric patients with Crohn's disease and induced a remission in 33% of them. Also, induction therapy with infliximab helps to increase body mass index. 

The possible mechanism of infliximab treatment improving mucosal healing has recently been studied. Kierkus et al. showed that a significant induction of regulatorymacrophageswas observed in patients with mucosal healing after treatment withinfliximab; while induction was absent in patients without mucosal healing. As shown in an in vitro model, the induced regulatory macrophages displayed the ability to induce wound healing, suggesting a key role forinfliximab-induced regulatory macrophages in mucosal healing [39]. 

### 3.7. Modulating Inflammatory Signal Pathways

Nuclear transcription factor-*κ*B (NF-*κ*B) is identified as the key regulator in immunological setting of IBD [75]. NF-*κ*B activation is markedly induced in IBD patients and through its ability to promote the expression of various proinflammatory genes, NF-*κ*B strongly influences the course of mucosal inflammation [76, 77]. TNF is one of the best-characterized agonists of NF-*κ*B pathway and is itself regulated by NF-*κ*B [75]. Turning down NF-*κ*B signals is another bioactivity of infliximab. Guidi et al. evaluated the production of TNF by peripheral blood mononuclear cells and the levels of NF-*κ*B family molecules in the intestinal mucosa during infliximab therapy in 12 patients [40]. Infliximab treatment increased NF-*κ*aB inhibitor levels including I*κ*B*α* and I*κ*B*γ*, and thus downregulated NF-*κ*B signals and TNF production in the intestinal mucosa of Crohn's disease patients [40]. 

However, another group found that infliximab enhances the activity and expression of mitogen-activated protein kinases (MAPKs) during the process of IBD. MAPKs are key signal pathways that cooperate in the orchestration of inflammatory responses, and extensive cross-talk to other inflammatory pathways, such as NF-*κ*B and Janus kinase/STAT signaling [78]. Four groups of MAPKs have been identified in mammalian cells: the extracellular signal-regulated kinases (ERKs), the c-Jun N-terminal kinases (JNKs) or stress-activated protein kinases (SAPKs), the p38 kinases, and ERK5/big MAPK [79, 80]. Waetzig et al. studied the activity and expression of the p38 MAPK alpha-delta, JNKs, and ERK1/2 in the inflamed intestinal mucosa of IBD patients [41]. Western blot analysis revealed that p38 alpha, JNKs, and ERK1/2 were significantly activated in IBD, with p38 alpha showing the most pronounced increase in kinase activity. In vivo inhibition of TNF by infliximab infusion resulted in a highly significant transient increase of p38 alpha activity during the first 48 h after infusion. A significant infliximab-dependent p38 alpha activation was also observed in THP-1 myelomonocytic cells. In human monocytes, infliximab enhanced TNF gene expression, which could be inhibited by SB 203580.

## 4. Genetic and Genomic Predictor of Infliximab Treatment

It was noted by clinical trials for IBD that initial response rate to infliximab treatment was only about 60%, and approximately 30% of the responders sustaining remission throughout 54 weeks [51]. Recently, the markers that predict response to infliximab treatment of IBD have been developed, such as clinical parameters including patient characteristics, smoking status and disease phenotype, and biological markers including C-reactive protein and serum TNF levels [81]. However, these markers are not so effective and stable to provide predictive value, and few markers have consistently replicated among clinical studies. For example, young age, smoking, and concomitant immunosuppressive treatment were reported as independent variables favoring short-term response to infliximab [82, 83], but no or reverse association was developed in other studies evaluating smoking and age [84, 85]. Development on new marker and factors with predictive value for IBD treatment is critical.

Genetic predictors of infliximab responsiveness become appealing, especially those candidate genes thought to participate in the pathogenesis or susceptibility of IBD. Hlavaty et al. reported that polymorphisms in FasL/Fas system and caspase-9 influenced the response toinfliximabin luminal and fistulizing Crohn's disease [86]. In the study, Fas ligand-843 TT genotype was presented exhibiting the strongest association with the nonresponse, while concomitant mercaptopurine/azathioprine therapy was linked with the effect of unfavourable genotypes in luminalCrohn's disease. To date, lots of candidate gene polymorphism was reported as potential predicators of anti-TNF response, including IBD5, TNF, TNF receptor, and NOD2 [81], but few gene markers showed high reproducibility among the studies. In the situation, combinations with different markers including genetic and nongenetic markers might be alternative option. A recent interesting study investigated multiple parameters to determine early response to infliximab in patients with ulcerative colitis [81]. In the study, disease activity, antineutrophil cytoplasmatic autoantibody (ANCA), and markers of inflammation were measured during 14 weeks infliximab infusion, and genotyping for IL23R gene variants was performed as well. Multivariate regression analysis identified high disease activity index before IFX therapy and negative ANCA status as independent positive predictors for response to infliximab. Homozygous carriers of inflammatory bowel disease (IBD) risk-increasing IL23R variants were more likely to respond to infliximab than were homozygous carriers of IBD risk-decreasing IL23R variants.

Genome wide association (GWA) predictors of infliximab responsiveness have been developed. Recently, 71 confirmed Crohn's disease susceptibility loci were reported by genome wide meta-analysis [87], and associations of the IBD susceptibility loci and novel “pharmacogenetic” GWAS identified loci with primary nonresponse to anti-TNF alpha in pediatric IBD patients were studied [88]. Dubinsky et al. determined associations of phenotype and genotype with primary nonresponse to infliximab infusion in both pediatric Crohn's disease and ulcerative colitis patients, and identified genetic associations by testing known IBD susceptibility loci and by performing a GWA for primary nonresponse [88]. They found that the combination of phenotype and genotype was most predictive of primary nonresponse to infliximab treatment in pediatric IBD. Defining GWA predictors of response to infliximab or other biological anti-TNF treatments may provide promise for clinical utility.

## 5. New Side Effects of Infliximab Treatment

Over the past 10 years, anti-TNF agents including infliximab have been emerging as an alternative treatment to overcome the shortcomings of conventional drugs and provide most of IBD patients the improved life quality. However, several side effects associated with infliximab have been recently reported (Table 2), including acute or delayed infusion reactions, leucopenia, serious infection, antichimeric antibody formation, and increased risk of malignancy [42–47, 89]. Steenholdt et al. showed that acute severe infusion reactions were strongly associated with development of anti-infliximab IgG antibody, and the risk was particularly high at the 2nd infusion in retreatment series [42]. Infliximabwas also reported to exacerbate clonal *γδ*-T cell expansion in vivo and induced *γδ*-T cell proliferation in vitro, indicating the potential risk of developing malignant *γδ*-T cell lymphomas following anti-TNF-*α* agent therapy [48].

It was also reported that infliximab treatment for IBD induced other autoimmune disease such as psoriasis. A Spanish group reported a 31-year-old woman with extensive Crohn's disease and perianal lesions developed a cutaneous eruption with psoriasiform morphology and distribution during treatment with both infliximab and adalimumab [49].

## 6. Conclusions

TNF, as the key proinflammatory cytokine during the development of IBD, plays the pivotal role in modulating mucosal immune response, including regulating immune cell proliferation, upregulating adhesion molecule expression, and inducing apoptosis of intestinal epithelial cells. By forming stable complexes with the human soluble or the membrane form of TNF expressed on cell surface, including immune cell, fibroblast, and epithelium, infliximab counteracts the activity of TNF, and plays an important role in IBD treatment. The known mechanism of infliximab is summarized in Table 1.

However, the biological action of infliximab on immune diseases is far from being well explored. For example, Th17 cells have been shown to participate in progress of IBD, but so far no evidence is available about impact of infliximab on Th17 cell function during the process of IBD. Infliximab has been thought to exert its biological functions through blocking TNF, while a recent study found that IL-15 and its soluble receptor might mediate the response to infliximab in patients with Crohn's disease [90]. Furthermore, infliximab is also clinically used for other immune diseases including arthritis, in which immune responses are not typically characterized by TNF [91, 92], in this way, we believe that infliximab is supposed to play biological action beyond neutralizing TNF production and counteracting TNF activity. 

## Figures and Tables

**Table 1:** Mechanisms of infliximab treatment on IBD.

Parameter	Effects	Ref.
Inflammatory cytokines, mediators	Downregulation	[18–21]
Activated lymphocytes	Apoptosis	[22–24]
Th1	Downregulation	[25–28]
Treg, regulatory macrophage	Induction	[29–31]
Intestinal epithelial cells and barrier	Protection	[32, 33]
ICAM1, VCAM1	Downregulation	[34]
Endothelial function	Protection	[35]
Mucosal angiogenesis	Inhibition	[36]
MMPs/TIMPs balance	Modulation	[37, 38]
Mucosa healing	Enhancing	[39]
NF-*κ*B signals	Inhibition	[40]
MAPKs signaling	Promotion	[41]

**Table 2:** Side effects of infliximab treatment.

Side effect	Ref.
Acute or delayed infusion reactions	[42]
Leucopenia	[43]
Serious infection	[42, 44, 45]
Antichimeric antibody formation	[46]
Increased risk of malignancy and lymphomas	[47, 48]
Other autoimmune diseases such as psoriasis	[49]

## References

[1] Xavier RJ, Podolsky DK (2007). Unravelling the pathogenesis of inflammatory bowel disease. *Nature*.

[2] Kamada N, Hisamatsu T, Okamoto S (2005). Abnormally differentiated subsets of intestinal macrophage play a key role in Th1-dominant chronic colitis through excess production of IL-12 and IL-23 in response to bacteria. *Journal of Immunology*.

[3] Bai AP, Ouyang Q (2006). Probiotics and inflammatory bowel diseases. *Postgraduate Medical Journal*.

[4] Papadakis KA, Targan SR (2000). Role of cytokines in the pathogenesis of inflammatory bowel disease. *Annual Review of Medicine*.

[5] Apostolaki M, Armaka M, Victoratos P, Kollias G (2010). Cellular mechanisms of TNF function in models of inflammation and autoimmunity. *Current Directions in Autoimmunity*.

[6] Khor B, Gardet A, Xavier RJ (2011). Genetics and pathogenesis of inflammatory bowel disease. *Nature*.

[7] Komatsu M, Kobayashi D, Saito K (2001). Tumor necrosis factor-*α* in serum of patients with inflammatory bowel disease as measured by a highly sensitive immuno-PCR. *Clinical Chemistry*.

[8] Stevens C, Walz G, Singaram C (1992). Tumor necrosis factor-*α*, interleukin-1*β*, and interleukin-6 expression in inflammatory bowel disease. *Digestive Diseases and Sciences*.

[9] Wang J, Fu YX (2005). Tumor necrosis factor family members and inflammatory bowel disease. *Immunological Reviews*.

[10] Nikolaus S, Raedler A, Kühbacher T, Sfikas N, Fölsch UR, Schreiber S (2000). Mechanisms in failure of infliximab for Crohn’s disease. *Lancet*.

[11] Rutgeerts P, Van Assche G, Vermeire S (2004). Optimizing anti-TNF treatment in inflammatory bowel disease. *Gastroenterology*.

[12] Sandborn WJ (2008). Current directions in IBD therapy: what goals are feasible with biological modifiers?. *Gastroenterology*.

[13] D’Haens G, Daperno M (2006). Advances in biologic therapy for ulcerative colitis and Crohn’s disease. *Current Gastroenterology Reports*.

[14] Rutgeerts P, Vermeire S, Van Assche G (2009). Biological therapies for inflammatory bowel diseases. *Gastroenterology*.

[15] Yamamoto-Furusho JK (2007). Innovative therapeutics for inflammatory bowel disease. *World Journal of Gastroenterology*.

[16] Danese S (2008). Mechanisms of action of infliximab in inflammatory bowel disease: an anti-inflammatory multitasker. *Digestive and Liver Disease*.

[17] Cornillie F, Shealy D, D’Haens G (2001). Infliximab induces potent anti-inflammatory and local immunomodulatory activity but no systemic immune suppression in patients with Crohn’s disease. *Alimentary Pharmacology and Therapeutics*.

[18] Atzeni F, Turiel M, Capsoni F, Doria A, Meroni P, Sarzi-Puttini P (2005). Autoimmunity and anti-TNF-*α* agents. *Annals of the New York Academy of Sciences*.

[19] Stokkers PCF, Hommes DW (2004). New cytokine therapeutics for inflammatory bowel disease. *Cytokine*.

[20] Ljung T, Axelsson LG, Herulf M, Lundberg JO, Hellström PM (2007). Early changes in rectal nitric oxide and mucosal inflammatory mediators in Crohn’s colitis in response to infliximab treatment. *Alimentary Pharmacology and Therapeutics*.

[21] Agnholt J, Kelsen J, Brandsborg B, Jakobsen NO, Dahlerup JF (2004). Increased production of granulocyte-macrophage colony-stimulating factor in Crohn’s disease—a possible target for infliximab treatment. *European Journal of Gastroenterology and Hepatology*.

[22] Van den Brande JMH, Braat H, van den Brink GR (2003). Infliximab but not etanercept induces apoptosis in lamina propria T-lymphocytes from patients with Crohn’s disease. *Gastroenterology*.

[23] Di Sabatino A, Ciccocioppo R, Cinque B (2004). Defective mucosal T cell death is sustainably reverted by infliximab in a caspase dependent pathway in Crohn’s disease. *Gut*.

[24] ten Hove T, Van Montfrans C, Peppelenbosch MP, Van Deventer SJH (2002). Infliximab treatment induces apoptosis of lamina propria T lymphocytes in Crohn’s disease. *Gut*.

[25] Mannon PJ, Fuss IJ, Mayer L (2004). Anti-interleukin-12 antibody for active Crohn’s disease. *The New England Journal of Medicine*.

[26] Andou A, Hisamatsu T, Okamoto S (2009). Dietary histidine ameliorates murine colitis by inhibition of proinflammatory cytokine production from macrophages. *Gastroenterology*.

[27] Kamada N, Hisamatsu T, Okamoto S (2008). Unique CD14^+^ intestinal macrophages contribute to the pathogenesis of Crohn disease via IL-23/IFN-*γ* axis. *Journal of Clinical Investigation*.

[28] Agnholt J, Kaltoft K (2001). Infliximab downregulates interferon-*γ* production in activated gut T-lymphocytes from patients with Crohn’s disease. *Cytokine*.

[29] Veltkamp C, Anstaett M, Wahl K (2011). Apoptosis of regulatory T lymphocytes is increased in chronic inflammatory bowel disease and reversed by anti-TNFalpha treatment. *Gut*.

[30] Ricciardelli I, Lindley KJ, Londei M, Quaratino S (2008). Anti tumour necrosis-*α* therapy increases the number of FOXP3^+^ regulatory T cells in children affected by Crohn’s disease. *Immunology*.

[31] Fleming BD, Mosser DM (2011). Regulatory macrophages: setting the threshold for therapy. *European Journal of Immunology*.

[32] Suenaert P, Bulteel V, Lemmens L (2002). Anti-tumor necrosis factor treatment restores the gut barrier in Crohn’s disease. *American Journal of Gastroenterology*.

[33] Zeissig S, Bojarski C, Buergel N (2004). Downregulation of epithelial apoptosis and barrier repair in active Crohn’s disease by tumour necrosis factor *α* antibody treatment. *Gut*.

[34] Arijs I, De Hertogh G, Machiels K (2011). Mucosal gene expression of cell adhesion molecules, chemokines, and chemokine receptors in patients with inflammatory bowel disease before and after infliximab treatment. *American Journal of Gastroenterology*.

[35] Schinzari F, Armuzzi A, De Pascalis B (2008). Tumor necrosis factor-*α* antagonism improves endothelial dysfunction in patients with Crohn’s disease. *Clinical Pharmacology and Therapeutics*.

[36] Scaldaferri F, Vetrano S, Sans M (2009). VEGF-A links angiogenesis and inflammation in inflammatory bowel disease pathogenesis. *Gastroenterology*.

[37] Meijer MJ, Mieremet-Ooms MAC, van Duijn W (2007). Effect of the anti-tumor necrosis factor-*α* antibody infliximab on the ex vivo mucosal matrix metalloproteinase-proteolytic phenotype in inflammatory bowel disease. *Inflammatory Bowel Diseases*.

[38] Di Sabatino A, Pender SLF, Jackson CL (2007). Functional modulation of Crohn’s disease myofibroblasts by anti-tumor necrosis factor antibodies. *Gastroenterology*.

[39] Kierkus J, Dadalski M, Szymanska E (2012). The impact of infliximab induction therapy on mucosal healing and clinical remission in Polish pediatric patients with moderate-to-severe Crohn's disease. *European Journal of Gastroenterology & Hepatology*.

[40] Guidi L, Costanzo M, Ciarniello M (2005). Increased levels of NF-kappaB inhibitors (IkappaBalpha and IkappaBgamma) in the intestinal mucosa of Crohn's disease patients during infliximab treatment. *International Journal of Immunopathology and Pharmacology*.

[41] Waetzig GH, Seegert D, Rosenstiel P, Nikolaus S, Schreiber S (2002). p38 mitogen-activated protein kinase is activated and linked to TNF-*α* signaling in inflammatory bowel disease. *Journal of Immunology*.

[42] Steenholdt C, Svenson M, Bendtzen K, Thomsen OA, Brynskov J, Ainsworth MA (2011). Severe infusion reactions to infliximab: aetiology, immunogenicity and risk factors in patients with inflammatory bowel disease. *Alimentary Pharmacology and Therapeutics*.

[43] Sherlock M, Bandsma R, Ota K, Kirby-Allen M, Griffiths A (2011). Severe neutropenia following infliximab treatment in a child with ulcerative colitis. *Inflammatory Bowel Diseases*.

[44] Veereman-Wauters G, de Ridder L, Veres G (2012). Risk of infection and prevention in pediatric patients with IBD: ESPGHAN IBD Porto Group commentary. *Journal of Pediatric Gastroenterology & Nutrition*.

[45] Schneeweiss S, Korzenik J, Solomon DH, Canning C, Lee J, Bressler B (2009). Infliximab and other immunomodulating drugs in patients with inflammatory bowel disease and the risk of serious bacterial infections. *Alimentary Pharmacology and Therapeutics*.

[46] Miele E, Markowitz JE, Mamula P, Baldassano RN (2004). Human antichimeric antibody in children and young adults with inflammatory bowel disease receiving infliximab. *Journal of Pediatric Gastroenterology and Nutrition*.

[47] Biancone L, Petruzziello C, Orlando A (2011). Cancer in Crohn’s disease patients treated with infliximab: a long-term multicenter matched pair study. *Inflammatory Bowel Diseases*.

[48] Kelsen J, Dige A, Schwindt H (2011). Infliximab induces clonal expansion of *γδ*-T cells in Crohn’s disease: a predictor of lymphoma risk?. *PLoS One*.

[49] Iborra M, Beltrán B, Bastida G, Aguas M, Nos P (2011). Infliximab and adalimumab-induced psoriasis in Crohn’s disease: a paradoxical side effect. *Journal of Crohn’s and Colitis*.

[50] Targan SR, Hanauer SB, Van Deventer SJH (1997). A short-term study of chimeric monoclonal antibody cA2 to tumor necrosis factor *α* for Crohn’s disease. *The New England Journal of Medicine*.

[51] Hanauer SB, Feagan BG, Lichtenstein GR (2002). Maintenance infliximab for Crohn’s disease: the ACCENT I randomised trial. *Lancet*.

[52] Desilva S, Kaplan G, Panaccione R (2008). Sequential therapies for Crohn’s disease: optimizing conventional and biologic strategies. *Reviews in Gastroenterological Disorders*.

[53] Swaminath A, Lichtiger S (2008). Dilation of colonic strictures by intralesional injection of infliximab in patients with Crohn’s colitis. *Inflammatory Bowel Diseases*.

[54] Taxonera C, Schwartz DA, García-Olmo D (2009). Emerging treatments for complex perianal fistula in Crohn’s disease. *World Journal of Gastroenterology*.

[55] Sands BE, Anderson FH, Bernstein CN (2004). Infliximab maintenance therapy for fistulizing Crohn’s disease. *The New England Journal of Medicine*.

[56] Rutgeerts P, Sandborn WJ, Feagan BG (2005). Infliximab for induction and maintenance therapy for ulcerative colitis. *The New England Journal of Medicine*.

[57] Sandborn WJ, Rutgeerts P, Feagan BG (2009). Colectomy rate comparison after treatment of ulcerative colitis with placebo or infliximab. *Gastroenterology*.

[58] Bamias G, Marini M, Moskaluk CA (2002). Down-regulation of intestinal lymphocyte activation and Th1 cytokine production by antibiotic therapy in a murine model of Crohn’s disease. *Journal of Immunology*.

[59] Hollander D (2002). Crohn’s disease, TNF-*α*, and the leaky gut. The chicken or the egg?. *American Journal of Gastroenterology*.

[60] Olsen T, Cui G, Goll R, Husebekk A, Florholmen J (2009). Infliximab therapy decreases the levels of TNF-*α* and IFN-*γ* mRNA in colonic mucosa of ulcerative colitis. *Scandinavian Journal of Gastroenterology*.

[61] Xu Y, Hunt NH, Bao S (2008). The role of granulocyte macrophage-colony-stimulating factor in acute intestinal inflammation. *Cell Research*.

[62] Mahida YR (2000). The key role of macrophages in the immunopathogenesis of inflammatory bowel disease. *Inflammatory Bowel Diseases*.

[63] Ogata H, Hibi T (2003). Cytokine and anti-cytokine therapies for inflammatory bowel disease. *Current Pharmaceutical Design*.

[64] Itoh J, de la Motte C, Strong SA, Levine AD, Fiocchi C (2001). Decreased Bax expression by mucosal T cells favours resistance to apoptosis in Crohn’s disease. *Gut*.

[65] Fuss IJ, Becker C, Yang Z (2006). Both IL-12p70 and IL-23 are synthesized during active Crohn’s disease and are down-regulated by treatment with anti-IL-12 p40 monoclonal antibody. *Inflammatory Bowel Diseases*.

[66] Bai A, Lu N, Guo Y, Chen J, Liu Z (2009). Modulation of inflammatory response via *α*2-adrenoceptor blockade in acute murine colitis. *Clinical and Experimental Immunology*.

[67] Kobayashi T, Okamoto S, Hisamatsu T (2008). IL23 differentially regulates the Th1/Th17 balance in ulcerative colitis and Crohn’s disease. *Gut*.

[68] Matsumura K, Nakase H, Yamamoto S (2009). Modulation of the Th1/Th2 balance by infliximab improves hyperthyroidism associated with a flareup of ulcerative colitis. *Inflammatory Bowel Diseases*.

[69] Hering NA, Schulzke JD (2009). Therapeutic options to modulate barrier defects in inflammatory bowel disease. *Digestive Diseases*.

[70] Schulzke JD, Bojarski C, Zeissig S, Heller F, Gitter AH, Fromm M (2006). Disrupted barrier function through epithelial cell apoptosis. *Annals of the New York Academy of Sciences*.

[71] Danese S, Sans M, de la Motte C (2006). Angiogenesis as a novel component of inflammatory bowel disease pathogenesis. *Gastroenterology*.

[72] Medina C, Radomski MW (2006). Role of matrix metalloproteinases in intestinal inflammation. *Journal of Pharmacology and Experimental Therapeutics*.

[73] Brew K, Nagase H (2010). The tissue inhibitors of metalloproteinases (TIMPs): an ancient family with structural and functional diversity. *Biochimica et Biophysica Acta*.

[74] Pineton De Chambrun G, Peyrin-Biroulet L, Lémann M, Colombel JF (2010). Clinical implications of mucosal healing for the management of IBD. *Nature Reviews Gastroenterology and Hepatology*.

[75] Atreya I, Atreya R, Neurath MF (2008). NF-*κ*B in inflammatory bowel disease. *Journal of Internal Medicine*.

[76] Hollenbach E, Neumann M, Vieth M, Roessner A, Malfertheiner P, Naumann M (2004). Inhibition of p38 MAP kinase- and RICK/NF-*κ*B-signaling suppresses inflammatory bowel disease. *The FASEB Journal*.

[77] Bantel H, Domschke W, Schulze-Osthoff K, Kaskas B, Gregor M (2000). Abnormal activation of transcription factor NF-*κ*B involved in steroid resistance in chronic inflammatory bowel disease. *American Journal of Gastroenterology*.

[78] Broom OJ, Widjaya B, Troelsen J, Olsen J, Nielsen OH (2009). Mitogen activated protein kinases: a role in inflammatory bowel disease?. *Clinical and Experimental Immunology*.

[79] Wilmanski JM, Petnicki-Ocwieja T, Kobayashi KS (2008). NLR proteins: integral members of innate immunity and mediators of inflammatory diseases. *Journal of Leukocyte Biology*.

[80] Hoffmeyer A, Grosse-Wilde A, Flory E (1999). Different mitogen-activated protein kinase signaling pathways cooperate to regulate tumor necrosis factor *α* gene expression in T lymphocytes. *Journal of Biological Chemistry*.

[81] Siegel CA, Melmed GY (2009). Predicting response to anti-TNF agents for the treatment of Crohn’s disease. *Therapeutic Advances in Gastroenterology*.

[82] Vermeire S, Louis E, Carbonez A (2002). Demographic and clinical parameters influencing the short-term outcome of anti-tumor necrosis factor (infliximab) treatment in Crohn’s disease. *American Journal of Gastroenterology*.

[83] Parsi MA, Achkar J, Richardson S (2002). Predictors of response to infliximab in patients with Crohn’s disease. *Gastroenterology*.

[84] Arnott IDR, McNeill G, Satsangi J (2003). An analysis of factors influencing short-term and sustained response to infliximab treatment for Crohn’s disease. *Alimentary Pharmacology and Therapeutics*.

[85] Fefferman DS, Lodhavia PJ, Alsahli M (2004). Smoking and immunomodulators do not influence the response or duration of response to infliximab in Chrohn’s disease. *Inflammatory Bowel Diseases*.

[86] Hlavaty T, Pierik M, Henckaerts L (2005). Polymorphisms in apoptosis genes predict response to infliximab therapy in luminal and fistulizing Crohn’s disease. *Alimentary Pharmacology and Therapeutics*.

[87] Franke A, McGovern DPB, Barrett JC (2010). Genome-wide meta-analysis increases to 71 the number of confirmed Crohn’s disease susceptibility loci. *Nature Genetics*.

[88] Dubinsky MC, Mei L, Friedman M (2010). Genome Wide Association (GWA) predictors of anti-TNF*α* therapeutic responsiveness in pediatric inflammatory bowel desease. *Inflammatory Bowel Diseases*.

[89] Parashette KR, Makam RC, Cuffari C (2010). Infliximab therapy in pediatric Crohn’s disease: a review. *Clinical and Experimental Gastroenterology*.

[90] Bouchaud G, Mortier E, Flamant M (2010). Interleukin-15 and its soluble receptor mediate the response to infliximab in patients with Crohn’s disease. *Gastroenterology*.

[91] Siddiqui MAA (2007). The efficacy and tolerability of newer biologics in rheumatoid arthritis: best current evidence. *Current Opinion in Rheumatology*.

[92] Gartlehner G, Hansen RA, Jonas BL, Thieda P, Lohr KN (2008). Biologics for the treatment of juvenile idiopathic arthritis: a systematic review and critical analysis of the evidence. *Clinical Rheumatology*.

